# Automated Counting of Cancer Cells by Ensembling Deep Features

**DOI:** 10.3390/cells8091019

**Published:** 2019-09-02

**Authors:** Qian Liu, Anna Junker, Kazuhiro Murakami, Pingzhao Hu

**Affiliations:** 1 Department of Biochemistry and Medical Genetics, University of Manitoba, Winnipeg, MB R3E 0J9, Canada; 2 European Institute of Molecular Imaging, University of Münster, D-48149 Münster, Germany; 3 Cancer Research Institute, Kanazawa University, Kanazawa 920 1192, Japan; 4 Research Institute in Oncology and Hematology, CancerCare Manitoba, Winnipeg, MB R3E 0V9, Canada

**Keywords:** microscopic imaging, automatic cell counting, deep learning, transfer learning, autoencoder, ensembling feature

## Abstract

High-content and high-throughput digital microscopes have generated large image sets in biological experiments and clinical practice. Automatic image analysis techniques, such as cell counting, are in high demand. Here, cell counting was treated as a regression problem using image features (phenotypes) extracted by deep learning models. Three deep convolutional neural network models were developed to regress image features to their cell counts in an end-to-end way. Theoretically, ensembling imaging phenotypes should have better representative ability than a single type of imaging phenotype. We implemented this idea by integrating two types of imaging phenotypes (dot density map and foreground mask) extracted by two autoencoders and regressing the ensembled imaging phenotypes to cell counts afterwards. Two publicly available datasets with synthetic microscopic images were used to train and test the proposed models. Root mean square error, mean absolute error, mean absolute percent error, and Pearson correlation were applied to evaluate the models’ performance. The well-trained models were also applied to predict the cancer cell counts of real microscopic images acquired in a biological experiment to evaluate the roles of two colorectal-cancer-related genes. The proposed model by ensembling deep imaging features showed better performance in terms of smaller errors and larger correlations than those based on a single type of imaging feature. Overall, all models’ predictions showed a high correlation with the true cell counts. The ensembling-based model integrated high-level imaging phenotypes to improve the estimation of cell counts from high-content and high-throughput microscopic images.

## 1. Introduction

To study the molecular mechanisms of complex diseases like cancer, microscopic images can provide valuable information, but it is often necessary to carry out a series of molecular biology experiments on several conditions [1,2]. Traditionally, the images from the experiments have been evaluated manually. Thus, it was time-consuming and needed a lot of human effort and expertise. Therefore, with the emerging development of high-content and high-throughput digital imaging systems, it is necessary to design new and automated analysis tools for current microscopic images. Among the variety of tasks done using microscopic images, cell counting is one of the crucial ones [3]. The number of cells in a microscopic image can be used as a measurement to be compared among different groups. For example, we can evaluate the treatment effect of a cancer drug at different doses by comparing the microscope-based cancer cell counts under the specified conditions [2]. Hence, the experimental group with the smallest cell count number in its microscopic images may be treated as the first dosage of the drug to be used for that specific cancer. The same principle can be applied to identify the most effective drugs as well [4]. Therefore, it is important for biologists to automatically collect accurate cell counts for each of the experimental groups under different experimental conditions so that further statistical significance can be modeled and evaluated.

In computer vision field, automated counting objects in static images have been mostly studied and practiced for crowd counting in pedestrian traffic to decrease traffic crash fatalities [5]. According to the review by Loy et al., there are two major strategies for automated object counting in static images: counting by detection and counting by regression [6]. Counting by detection was the earliest method used in object counting. It involves first training an object detector, then applying the detector to the whole image using a sliding window or other segmenting technique to identify the objects and estimate the number of objects [5,7,8]. Many detectors have been proposed and evaluated in different studies, but the performance of these detectors still has room for improvement, especially when the resolutions of the images are low and some of the objects in the images are overlapped [5,7]. Counting by regression is considered to be a more accurate and faster strategy [6]. A typical counting by regression study first employs a preprocessing step to extract low-level features like size, area, histogram, and texture, and high-level features like object foreground segment map, dot density representative map, etc., and then regresses these features to the object counts [8,9]. The preprocessing step can be implemented manually or automatically. A lot of efforts have been made to extract robust features automatically. For example, artificial neuron network (ANN), a well-studied machine learning algorithm inspired by the human neuron network, can be involved in both the feature extraction step and the regression step, and has been confirmed to have good performance on both tasks [10].

Among the features used in previous object counting studies, foreground masks and dot density maps have gained more and more attention in recent years due to the rapid development of deep artificial neural network (DANN) and autoencoder techniques [11]. DANN is a kind of special ANN with more hidden nodes and layers in its architecture, and it is also known as deep learning (DL) [12]. Autoencoder refers to a model strategy which usually consists of a contracting block to compress data from the input layer into a short code and a symmetric decoding block which enables that short code to be decompressed into something that closely matches the original data. For the two popular types of features mentioned above, a foreground mask is a kind of white and black binary image. Pixels in the objects (e.g., cancer cells) in the images are white (foreground), while pixels of the background are black [13]. Dot density maps aim to specify object position by putting a single dot on each object instance in each image [9]. These two types of features themselves can be plotted out as images and have the same sizes as their original images. Hence, using them as annotations or labels requires us to build powerful autoencoder-like architectures. Fortunately, the emerging DANN-based autoencoder technology provides us with more options.

By introducing a convolution operation into a DANN model, the DANN is turned into a deep convolutional neural network (DCNN). DCNN is widely used in computer vision due to its power in handling structured data like image data, and it has been applied to cell counting tasks. Xie et al. designed several fully connected DCNN models to first predict the dot density map, then sum the dots in the map up as the estimated count in a microscopic image [14]. The advantage of the density-based method is that it has the potential to address cell overlapping. However, this method is sensitive to the variances of datasets. When applying the algorithm to a varietal dataset rather than the one similar to the training set, extra fine-tuning work is necessary. Hernández et al. built a DCNN-based autoencoder named feature pyramid network (FPN) to generate foreground feature masks of microscopic images, then applied another DCNN model to regress the mask to cell count [10]. This strategy is more direct and does not need extra sum-up and fine-tuning steps for different datasets, but this kind of segmentation-based method cannot overcome limitations such as cell clumping and overlap [7].

To overcome these challenges, this study introduced a novel model architecture by first extracting both dot density map and foreground mask as imaging features, then stacking the two types of features together using a two-input DCNN model to regress them on the cell counts. This idea came from ensemble learning, which has been well studied and shown to achieve better prediction performance by using multiple learning algorithms [15,16,17]. There are several methods to assemble models; the method used in this study was called stacking learning, which employs a learning algorithm to combine the predictions of other algorithms. Each of these predictions was generated from all training data [18,19].

## 2. Materials and Methods

### 2.1. Datasets and Preprocessing Analysis

Three image datasets were used in this study (Table 1). Two of them were publicly available simulated microscopic images [13,20], which were well labeled with true cell counts and acted as the training datasets for feature extraction using our DCNN models. The other dataset was a real microscopic image dataset, which was generated in-house to evaluate the performance of our proposed models.

#### 2.1.1. Simulated Data

The two simulated datasets provided images in different formats and both had two types of annotations: the feature label and the cell count label. The dataset used for dot-density-map-based feature extraction contained 200 synthetic microscopic images (RGB) and their corresponding dot maps (gray images) with a resolution of 256 × 256. The average cell count number in each image was 174 ± 64. The dataset used for foreground-mask-based feature extraction contained 1200 gray-level synthetic microscopic images, and their corresponding foreground mask had a resolution of 696 × 520. The number of cells in the images varied from 1 to 100. To keep the two datasets under the same dimension, we reduced the three RGB channels of the images from the first dataset into one channel (i.e., transformed the RGB images to gray-level images) and resized the images in the second dataset to the resolution of 256 × 256.

#### 2.1.2. Real Data

We generated three different colorectal cancer organoids [2]: AKTP organoids (organoids with mutations in four cancer driver genes, *APC∆716* mutation/*KRasG12D* mutation/*TgfbrII* knock-out/*P53R270H* mutation) worked as a control group, while AKTP-P2rX7 organoids (AKTP organoids with *P2rX7* gene overexpression) and AKTP-Nt5e organoids (AKTP organoids with *Nt5e* gene overexpression) worked as treatment groups. Biologically speaking, *P2rX7* gene and *Nt5e* gene expressions could change the character of cells in AKTP organoids. If they enhance cell proliferation, cell number in each organoid should be increased. On the other hand, if *P2rX7* and *Nt5e* expressions affect cell–cell adhesion, such as induction of epithelial–mesenchymal transition (EMT) etc., the cell number in each organoid could be decreased. Basically, this experiment aimed to test the cell count differences between AKTP-P2rX7 organoids and AKTP organoids, and AKTP-Nt5e organoids and AKTP organoids using microscopic images generated from these three conditions. To do this, the AKTP organoids were cultured from the small intestine of a *APC∆716/KRasG12D/TgfbrII KO/p53R270H* colorectal cancer mouse model according to the standard protocol [21]. To overexpress *P2rx7* and *Nt5e*, we cloned mouse *P2rx7* or *Nt5e* genes into a mammalian expression vector which has a constitutive active promoter. We then introduced those plasmids into AKTP organoids and established two sets of organoids, making three different groups defined as AKTP, AKTP-P2rX7, and AKTP-Nt5e. To image them, these three groups of organoids were collected from Matrigel with cell recovery solution (Corning, NY, USA) and fixed with 4% paraformaldehyde (Sigma Aldrich, 158127) for 10 min at room temperature. Nuclei were then stained with Hoechst33342 (ThermoFisher Scientific (Waltham, MA, USA) for 10 min at room temperature. The stained organoids were placed onto glass slides and cover slipped using a mounting medium (Vectashield Laboratories (Burlingame, CA, USA. H-1000). To make the 3D organoids become 2D, we pressed the cover slip carefully before sealing it. After all the preparation processing, the slides were flatbed and scanned using a Leica TCS SP8 confocal microscope (Leica Microsystems, Wetzler, Germany). The view fields were digitized at 20× magnification (20× objective) and images were processed using Leica Application Suite X software (Leica Microsystems). To reduce noise, an average image was taken from two images. We did not take Z-stacks, as organoids were 2D on the glass slides. Therefore, each image contained all nuclei in an organoid. In total, we gathered 164 images for the AKTP group, 107 images for the AKTP-P2rX7 group, and 114 images for the AKTP-Nt5e group from the experiment. These images had a resolution of 512 × 512, so we resized them to the resolution of 256 × 256 to make them the same resolution as the datasets used in the training models.

### 2.2. Models

**Figure 1:**
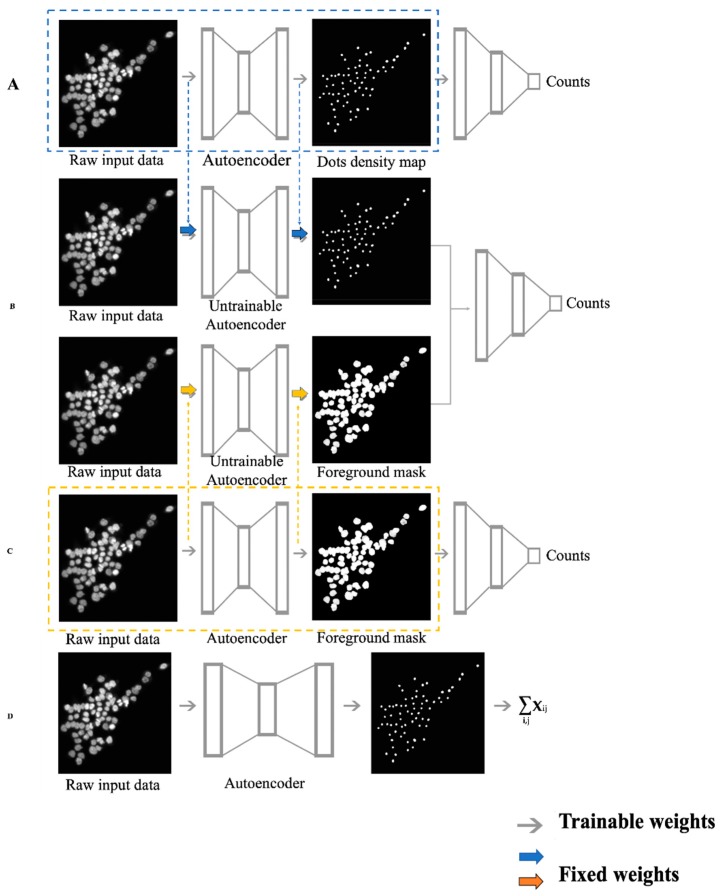
The workflow of the four deep convolutional neural network (DCNN) models used in this study. Gray arrows are trainable weights in the models. Colored arrows represent the weights inherited from previous well-trained feature extraction models. (**A**) Dot-density-map-based regression DCNN (DRDCNN). It contained two parts: feature extraction autoencoder (dashed box) and regression parts. The blue dashed arrows represent the well-trained weights, which can be loaded into the end-to-end counting regression model. (**B**) Ensembling-based regression DCNN (ERDCNN). Colored arrows represent the weights inherited from the two previous well-trained feature extraction models. (**C**) Foreground-mask-based regression DCNN (FRDCNN). It contained feature extraction autoencoder and regression parts as well. The orange arrows represent the well-trained weights which can be transferred into the ensemble regression model. (**D**) Density-only DCNN (DDCNN). This model first trained an autoencoder to extract a dot density map from a microscopic image, then summed the pixel values up in the dot density map to get the estimated cell count without any regression process.

Since a lot of similar feature extraction models [10,22] have been trained in previous studies and were reported to have decent performance, we inherited their essences in a transfer learning way by borrowing their well-trained parameters to initialize our proposed models (Figure 1). Transfer learning emerged to address the insufficient label issue in the machine learning field [23] and was shown to have excellent ability to enhance the learning powers of DANNs [24]. Since our real data had no true labels or cell counts, our proposed ensembling model was built on the concept of transfer learning. We first trained the two feature-extraction-based DCNN autoencoder models (dot density map regression DCNN (DRDCNN, Figure 1A) and foreground mask regression DCNN (FRDCNN, Figure 1C) on the two publicly available and well-labeled datasets; we then borrowed the well-trained weights into our proposed ensembling-based regression DCNN model (ERDCNN, Figure 1B), which had similar architecture to the two feature extraction models in the first half. After the trained weights were loaded into the ERDCNN model, we fixed them and set only the remaining layers as trainable, and then we further trained the ERDCNN model using the combined dataset. Finally, we fixed all parameters and applied the trained model to predict the cell counts in our real microscopic images, and performed differential analysis of the estimated cell counts under different conditions. The three DCNN models were developed using Keras [25] with Tensorflow [26] as backend. The DRDCNN model and FRDCNN model were built to find the well-trained weights by using autoencoder-like architectures to extract the known dot density maps and foreground masks, respectively, from the original microscopic images. They also acted as baseline models in this study for us to compare the performance of the proposed ERDCNN model.

In addition, we also employed Xie’s U-net-based density-only deep convolutional neural network model (DDCNN, Figure 1D) [14] as the third baseline model for our proposed ensembling-based regression DCNN model. This model directly sums up the pixel values of the predicted dot density map as the estimated cell count instead of having a regression tail.

#### 2.2.1. Dot Density Map Regression DCNN Model (DRDCNN)

This model was composed of a U-net [27] and a VGG (Visual Geometry Group) [28] tail. The U-net part acted as an autoencoder to regress the original image to its dot density map, while the VGG tail was used to collapse the dot density map into its cell count. The U-net is a DCNN architecture well-known due to its great performance in the microscopic image cell tracking challenge [27]. For the regression tail, we generated it using a simplified VGG strategy by stacking four convolutional blocks with two dense layers. The convolutional block had a batch normalization layer, a convolutional layer with very small (3 × 3) convolutional filters activated by a leaky ReLu (rectified linear unit) function [29], a dropout layer with the proportion of dropout set as 0.6, and a max pooling layer. This architecture has been shown to have better performance in cell counting regression [10]. Among the technologies listed above, batch normalization and dropout were intended to address the parameter initialization and overfitting problems by normalizing each training mini-batch [30] and dropping out a certain proportion of output from previous layers [31]. Using a small filter size (3 × 3) instead of other sizes of filter (7 × 7, 5 × 5) is the key development of VGG-like structure, since it generates stable and robust representatives even when the DCNN goes deeper [28].

We designed two steps for training this model because we had two levels of annotations or labels which had different purposes. Before the training start, 150 of the 200 images were randomly assigned to a training set, while the remaining 50 images plus another 50 images from the second simulated dataset were put into a common test set (Table 2). In the first training step, we borrowed the well-trained weights from Xie et al. [14] in a transfer learning way. The weights were downloaded from their website and loaded into our U-net part as the initialization of our autoencoder. The dot density maps were then given as labels to the U-net. Adam was selected as the optimizer and loss function was set to MSE (mean square error). The parameters of epoch and batch size were set to 192 and 16, respectively. After this step, our U-net and its well-trained weights were saved into a json file and an hd5 file separately to build the ERDCNN model (see Section 2.2.3). The weights were then fixed so that we could move to the second step of the training. In the second step, the cell counts were used as labels and the original images were still used as input data. Instead of using the 150 images in the first training step, the combined 1300 images from the two simulated datasets (there were 150 and 1150 images from the first and the second simulated datasets, respectively) were used as the training set in this step (Table 2). In this way, the whole model was actually still end-to-end. Since the autoencoder part was fixed, only the regression tail was trainable, which saved training time. Epoch was set to 1000. The main aim of the second step was to get the predicted cell count for each image in the test set as one of the benchmarks of the prediction results from the proposed model discussed in Section 2.2.3. This model is summarized in Algorithm 1. It should be noted that a previous version of the algorithm has been published by our group [32]. However, in the previous study, we tested the DRDCNN model using the dot density map dataset only, while here, we tested it using both the dot density map dataset and the foreground mask dataset.

**Algorithm 1** DRDCNN modelInput:  two training sets: *S1*, *S2*;                 test set: *T*;
             models: U-net (*U*), VGG (*V*);
             transferred weights from the published study: *W*                 dot density maps: *D_S1_*;                 cell counts label: *C_concatenate(S1, S2)_*.Procedure:          *1.*   *L*1 = *f*_1_ (*U* (*W_u_*, *S1*), *D_S1_*))                 train a set of weights *W_1_* which was initialized by *W_u_* to minimize the loss function *L1*.             *2*. *L*2 = *f*_2_ (*V* (*W*_2_, *U* (*W*_1_, *concatenate* (*S*1, *S*2)), *C_concatenate(S1, S2)_*))             *W*_1_ was initialized from Step 1 and fixed to untrainable.               *W*_2_ was trained to minimize the loss function *L*2.
Output:  predicted cell counts: *c_DRDCNN_*.                       *c_DRDCNN_* = *V* (*W*_2_, *U* (*W*_1_, *T*))

#### 2.2.2. Foreground Mask Regression DCNN Model (FRDCNN)

Very similar to the DRDCNN, the FRDCNN model also consisted of an autoencoder to regress the original images to their foreground masks and a VGG-style regression tail to collapse those masks to their cell counts. Instead of borrowing the weights from the U-net, we generated a basic convolutional autoencoder with three convolutional layers followed by three max pooling layers as an encoder, and three convolutional layers followed by three up-sampling layers as a decoder.

The strategy used to train the model was a little different from the DRDCNN. Table 2 details the utilization of the datasets. Briefly, 1150 of the 1200 images in the second simulated dataset were assigned to a training set, which left us 50 images for the common test set (100 images in total). Since there was no transfer learning in the first step, we initialized the weights using the Keras default orthogonal method. Other parameters were the same as those used by the DRDCNN. Once the loss was converged, we saved the autoencoder structure and its well-trained weights for building the ERDCNN in Section 2.2.3. The FRDCNN is summarized in Algorithm 2.

**Algorithm 2** FRDCNN modelInput:  two training sets: *S*1, *S*2;                test set: *T*;
           models: self-built autoencoder (*A*), VGG (*V*);
           transferred weights from the published study: *W_u_*                    foreground masks: *M_S2_*;                    cell counts label: *C_concatenate(S1, S2)_*.Procedure:             *1.*    *L3* = *f_3_*(*A* (*W_3_*, *S2*), *M_S2_*))               train a set of weights *W_3_* which was initialized randomly to minimize the loss function *L3*.             *2.*   *L*4 = *f*_4_ (*V* (*W*_4_, *A* (*W*_3_, *concatenate* (*S*1, *S*2)), *C*_*concatenate*(*S*1, *S*2)_))                  *W*_3_ was initialized from Step 1 and fixed to untrainable.                   *W*_4_ was trained to minimize the loss function *L4*.
Output:  predicted cell counts: *c_DRDCNN_*.                   *c_FRDCNN_* = *V* (*W*_2_, *U* (*W*_1_, *T*)

#### 2.2.3. Ensembling Concatenated Regression DCNN model (CRDCNN)

The ERDCNN has two branches at the beginning of its architecture, as shown in Figure 1. One is the autoencoder for extracting dot density maps using the U-net, which were the output from DRDCN model, while the other is the autoencoder for extracting foreground masks, which were the output from FRDCNN model. The decoded outputs of these two branches were concatenated in the middle of the architecture. The concatenated features were then input into the same structured regression tail to predict the cell count for each microscopic image.

To train this model, we merged the two training sets mentioned above to get a larger training set (1300 images) and kept the same common test set with 100 images to get predicted cell counts (Table 2). In this way, we ensured that the predictions from the three models were based on the same test set to make them comparable. Two well-trained weight sets saved from the previous models (Section 2.2.1 and Section 2.2.2) were then loaded into these two branches accordingly, and were set to be untrainable. Cell counts were used as labels for the backpropagation training procedure. The ERDCNN model is summarized in Algorithm 3.

**Algorithm 3** CRDCNN modelInput:  two training sets: *S1*, *S2*;                test set: *T*;
           models: U-net (*U*), self-built autoencoder (*A*), VGG (*V*);
            dot density maps: *D_S1_*;                foreground masks: *M_S2_*                cell counts label: *C_concatenate(S1, S2)_*Procedure:             *1.*   *L*5 = *f*_5_ (*V* (*W*_5_, *concatenate* (*U* (*W*_1_, *concatenate* (*S*1, *S*2)), *A* (*W*_3_, *concatenate* (*S*1, *S*2))), *C*_*concatenate*(*S*1, *S*2)_))                  *W*_1_ was transferred from the DRDCNN model and fixed to untrainable.                    *W*_3_ was transferred from the FRDCNN model and fixed to untrainable.                   *W*_5_ was trained to minimize the loss function *L*5.
Output:  predicted cell counts: *c_DRDCNN_*.             *c_ERDCNN_* = *V* (*W*_5_, *concatenate* (*U* (*W*_1_, *T*), *A* (*W*_3_, *T*)))

#### 2.2.4. Performance Evaluation

Since the cell counting was treated as a regression problem in this study, the final target variable (cell count) was a continuous numeric. We evaluated the performances of the models by looking at how much the predictions deviated from the actual cell counts on average.

There are four commonly used evaluation metrics available for such regression models: root mean square error (*RMSE*), mean absolute error (*MAE*), mean absolute percent error (*MAPE*), and the Pearson correlation (*r*). RMSE represents the sample standard deviation of the differences between the predicted values and true values. Mathematically, it is calculated using Equation (1). *MAE* is the averaged absolute difference between the predicted values and true values. It is calculated using Equation (2). *MAPE* is calculated with Equation (3), and it represents the mean absolute percent difference between two numeric vectors, while *r* is the standard Pearson correlation coefficient as calculated using Equation (4).

(1) $$RMSE\;=\;\sqrt{mean\;({\left(c-\hat{c}\right)}^{2})}$$

(2) $$MAE\;=\;mean\;(\left|c-\hat{c}\right|)$$

(3) $$MAPE\;=\;mean\;(\frac{c-\hat{c}}{\left|c\right|})$$

(4) $$r=\frac{\sum_{i=1}^{n}\left(x_{i}-\bar{x}\right)\left(y_{i}-\bar{y}\right)}{\sqrt{\sum_{i=1}^{n}{\left(x_{i}-\bar{x}\right)}^{2}}\sqrt{\sum_{i=1}^{n}{\left(y_{i}-\bar{y}\right)}^{2}}}$$

where *c* = [*x*_1_, *x*_2_, ..., *xn*] represents the true values of the cell counts for the n images in the test set, and $\hat{c}$ = [*y*_1_, *y*_2_, ..., *yn*] represents the predicted cell counts for the n images in the test set. An error metric of zero indicates that the model fits the data perfectly. By contrast, a higher value of r indicates a better fit of the model.

#### 2.2.5. Real Data Application

Once the models were all well-trained and all parameters and weights were fixed, models were then used to predict the cell counts for our real microscopic image datasets generated in the three conditions: 164 images from AKTP organoids, 107 images from AKTP-P2rX7 overexpressed organoids, and 114 images from AKTP-Nt5e overexpressed organoids. A *t*-test was then performed to test the difference of the predicted cell counts between AKTP vs. AKTP-P2rX7 and between AKTP vs. AKTP-Nt5e.

## 3. Results

**Figure 2:**
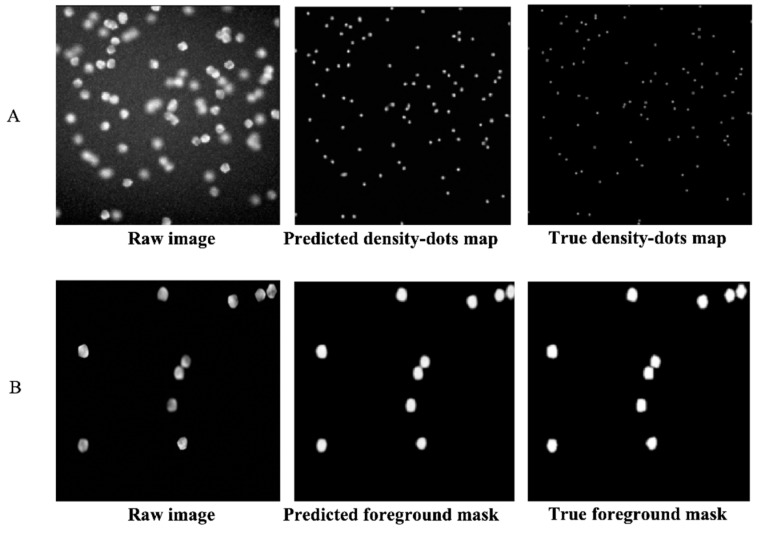
Features predicted by autoencoders of DRDCNN and FRDCNN. (**A**): Predicted dot density map by the autoencoder part of DRDCNN. The predicted one (in the middle) was a little brighter than the true dot density map but had the full ability to represent the center of each cell in the raw image. (**B**): Predicted foreground mask by the autoencoder part of FRDCNN. The predicted foreground mask was smoother than the true mask, but this small drawback did not influence its representative for the foreground of the raw image.

After the first training step of the DRDCNN and FRDCNN (i.e., the training of the autoencoders), predictions were made as to whether the autoencoders could achieve the purpose of feature extraction. The loss of the DRDCNN-based autoencoder was converged to around 0.7, while the loss of the FRDCNN-based autoencoder was converged to around 10. Figure 2 shows the raw images, predicted features, and the label features (ground truth). Both the predicted dot density map (Figure 2A) and the predicted foreground mask (Figure 2B) were quite similar to the true ones. Hence, they had the full ability to represent the features we expected from the raw image data.

**Figure 3:**
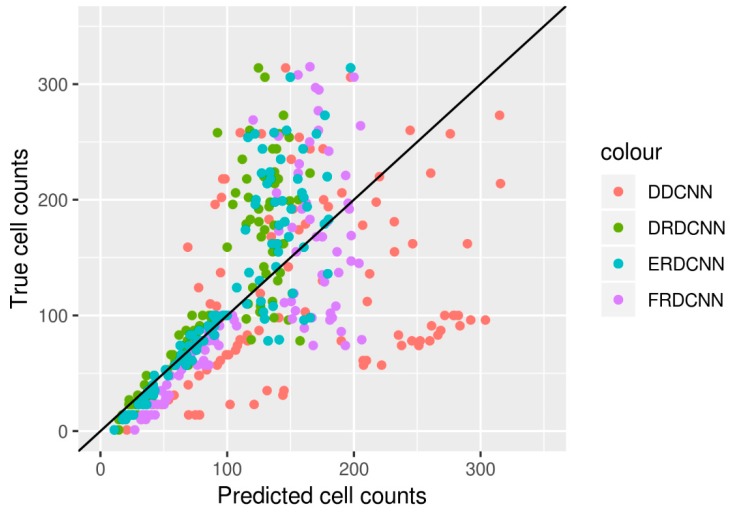
Cell counts predicted by the four models vs. their true cell counts based on the 100 test images. Predictions made by the ERDCNN model were closer to the true cell counts; especially for those images with smaller cell counts (e.g., the number of cells is smaller than 100).

We then evaluated the performance of the four end-to-end DCNN models by predicting the cell counts of the test set with the 100 images. The results are shown in Table 3, and the three regression-based DCNNs’ predictions (DRDCNN, FRDCNN, and ERDCNN) are further shown in Figure 3. The proposed ensembling-based regression DCNN model (ERDCNN) had better performance in terms of lower errors (*RMSE* = 49.25; *MAE* = 31.49) and higher correlation (*r* = 0.85).

**Figure 4:**
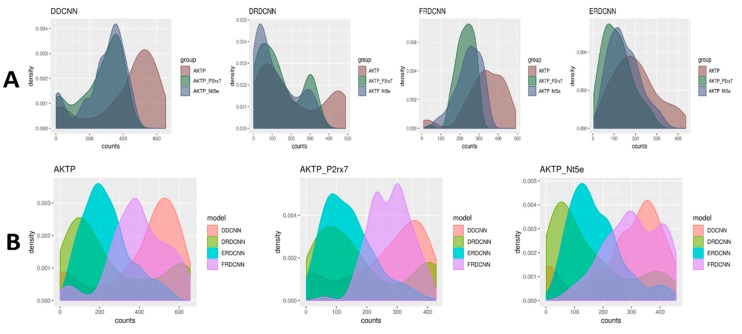
Density plots of the predicted cell counts for the three experimental groups from the four models. (**A**) Each plot compared the predictions of the three experimental groups using the same model. (**B**) Each plot compared the predictions of the four models for the same experimental group.

The trained models were used to predict the cell counts for the microscopic images generated from the three experimental groups (where AKTP was the control group, AKTP-P2rx7 and AKTP-Nt5e were the case groups). The t-test results comparing the predicted cell count differences between the control group and the case groups are shown in Table 4. There was a significant difference of the predicted cell counts between the AKTP-P2rx7 group and the AKTP control group by the ERDCNN and FRDCNN models, where P-values of the ERDCNN, FRDCNN, and DDCNN were 0.0002, 0.002, and 0.009, respectively. The predicted mean cell count by the ERDCNN in the AKTP-P2rx7 group (24.26) was lower than that in the AKTP group (29.69), suggesting that P2rx7 over-expression induced the EMT phenotype in the malignant colorectal cancer organoids and the EMT phenotype inhibited the cell growth of those organoids. However, there was no difference in the predicted cell counts by the ERDCNN between the AKTP-Nt5e group (29.80) and the AKTP group (29.69). A similar conclusion of no significant difference of the predicted cell counts between the AKTP-Nt5e group and the AKTP group was also observed using either the DRDCNN or the FRDCNN model. However, the DDCNN model suggested decreasing cell counts in the AKTP-Nt5e group compared to in the control group (*p*-value = 0.03). The distributions of the cell count predictions were very diverse (Figure 4), which indicated that the models might not be able to predict the exact number of cells in an image from the datasets other than the set under which these models were trained. However, they (e.g., ERDCNN and FRDCNN) were able to predict the overall cell count difference.

## 4. Discussion

In this study, we developed an integrated end-to-end DCNN model (ERDCNN) to regress microscopic image features to image-specific cell counts. The model integrated the DRDCNN model, which had a U-net as feature extractor, and the weights were initialized from a previous study, and the FRDCNN model, which had a VGG-style autoencoder as feature extractor. Except for the difference in the feature extraction step, the three regression models were quite similar in terms of having a common convolutional regression tail. We also compared the performance of the new model with the published density-only DCNN (DDCNN) model and the two regression-based models (DRDCNN and FRDCNN).

Two synthetic datasets were used in the training and testing procedures. We designed a training strategy by integrating transfer learning of the model weights and ensembling the learned image features. The highest correlation between the true cell counts and the predicted cell counts of the 100 test images was found using the proposed ERDCNN model. The well-trained models were applied to estimate the cell counts of the real microscopic images generated in three experimental conditions. A significant difference of the estimated cell counts was found between one of the two case conditions and the control condition using the proposed model. Although we observed improved performance in our proposed new model (ERDCNN), it took much longer to train the model (1 h for DRDCNN and FRDCNN vs. 5 h for ERDCNN) on a GPU-based Nvidia GeForce GTX 1080 with an 8 GB memory machine.

We observed a major decrease in the performance of the models when the number of cell counts was larger than 100. The major reason for the abnormality was due to the nature of the dataset used in the study. The data were combined from two different data sources, which varied in size, dimension, depth, and, more importantly, cell counts. In one source, the counts ranged from 1 to 100, while in the other source, they ranged from 1 to around 200. The unbalanced distributions of the two dataset sources might have caused the tremendous change of the performance. Another issue might be that the two data sources were generated using different algorithms. To overcome the challenges as much as possible, we did a series of preprocessing, such as resizing, reshaping, and rescaling, to make a combined dataset meeting the requirements of the both branches. The ideal situation would have been to simulate a whole dataset with both density map and foreground mask as labels so that we did not have to combine two datasets together. However, this was outside the scope of this research, and we will explore this issue in more detail in the future.

The model may be improved by incorporating more high-level image features, since our investigation of ensembling two types of image features instead of one type of feature improved the model’s prediction ability. Another limitation of this study was the quality of the data. We only had two synthetic datasets which were generated by different algorithms and had different sizes, dimensions, depths, and, more importantly, cell counts. The cell counts in the images were distributed sparsely. Each cell count category had only one or a few samples, which may be far from enough for a deep regression model like the one we developed here.

In summary, we proposed a strategy to ensemble high-level image features for cell counting, and showed that including more image features would increase the performance and stability of the DCNN model in cell counting.

## Figures and Tables

**Table 1:** The two synthetic datasets.

Datasets		Dataset Profiles		
		Size of Images	Resolution	Channel
Dot density	Raw data	200	256 × 256	3
	Preprocessed data	200	256 × 256	1
Foreground	Raw data	1200	696 × 520	1
	Preprocessed data	1200	256 × 256	1
Real data	Raw data	385 *	512 × 512	1
	Preprocessed data	385	256 × 256	1

* 164 of AKTP organoids (organoids with mutations in four cancer driver genes, *APC∆716* mutation/*KRasG12D* mutation/*TgfbrII* knock-out/*P53R270H* mutation), 107 of AKTP-P2rX7 (AKTP organoids with *P2rX7* gene overexpression) knock down organoids, and 114 of AKTP- Nt5e (AKTP organoids with *Nt5e* gene overexpression) knock down organoids.

**Table 2:** The three regression DCNN models.

			DRDCNN		FRDCNN		ERDCNN	
			Autoencoder	Regression	Autoencoder	Regression	Autoencoder	Regression
Dataset 1 ^1^	Training set (*n* = 150)	Raw image	√ ^3^	√	× ^4^	√	×	√
		Feature label	√	×	×	×	×	×
		Count label	×	√	×	√	×	√
	Testing set (*n* = 50)	Raw image	√	√	×	√	×	√
		Feature label	√	×	×	×	×	×
		Count label	×	√	×	√	×	√
Dataset 2 ^2^	Training set (*n* = 1150)	Raw image	×	√	√	√	×	√
		Feature label	×	×	√	×	×	×
		Count label	×	√	×	√	×	√
	Testing set (*n* = 50)	Raw image	×	√	√	√	×	√
		Feature label	×	×	√	×	×	×
		Count label	×	√	×	√	×	√

^1^ The dataset with 200 synthetic microscopic images and their corresponding dot density maps as well as the cell counts. Training set with 150 samples and testing set with 50 samples were randomly split. ^2^ The dataset with 1200 synthetic microscopic images and their corresponding foreground masks as well as the cell counts. Training set with 1150 samples and testing set with 50 samples were randomly split. ^3^ √ indicates that the set was used to train the certain part of the model. ^4^ × indicates that the set was not used to train the certain part of the model.

**Table 3:** Performance of the four models on the 100 test images.

	DDCNN	DRDCNN	FRDCNN	ERDCNN
*RMSE*	98.24	57.68	56.07	49.25
*MAE*	78.65	37.49	39.48	31.49
*MAPE*	1.32	0.33	0.35	0.28
*r*	0.34	0.81	0.74	0.85

**Table 4:** Results of the t-tests evaluating the predicted cell counts under the three different conditions using the different DCNN models.

		DDCNN	DRDCNN	FRDCNN	ERDCNN
AKTP-P2rx7 vs. AKTP	*p*-value	0.0009	0.84	0.002	0.0002
	Mean (x,y)	5.13, 6.66	16.70, 16.93	44.89, 53.49	24.26, 29.69
AKTP-Nt5e vs. AKTP	*p*-value	**0.03**	0.27	0.52	0.94
	Mean (x,y)	6.67, 5.99	17.81, 16.93	55.73, 53.49	29.80, 29.69
